# Comprehensive analyses of somatic *TP53* mutation in tumors with variable mutant allele frequency

**DOI:** 10.1038/sdata.2017.120

**Published:** 2017-09-05

**Authors:** Alexander J. Cole, Ying Zhu, Trisha Dwight, Bing Yu, Kristie-Ann Dickson, Gregory B. Gard, Jayne Maidens, Susan Valmadre, Anthony J. Gill, Roderick Clifton-Bligh, Deborah J. Marsh

**Affiliations:** 1 Hormones and Cancer Group, Kolling Institute of Medical Research, Royal North Shore Hospital, University of Sydney, New South Wales 2065, Sydney, Australia; 2 Hunter New England Health, New South Wales 2305, Australia; Royal North Shore Hospital, St Leonards, New South Wales 2065, Australia; 3 Department of Medical Genomics, Royal Prince Alfred Hospital, Sydney, New South Wales 2050, Australia; 4 Sydney Medical School, University of Sydney, Sydney, New South Wales 2006, Australia; 5 Department of Obstetrics and Gynaecology, Royal North Shore Hospital, St Leonards, New South Wales 2065, Australia; 6 Mater Private and Royal North Shore Hospitals, Sydney, NSW 2065, Australia; 7 Department of Anatomical Pathology, Royal North Shore Hospital, University of Sydney, Sydney, New South Wales 2006, Australia; 8 Cancer Diagnosis and Pathology Research Group, Kolling Institute of Medical Research, Royal North Shore Hospital, St Leonards, New South Wales 2065, Australia

**Keywords:** Diagnostic markers, Tumour biomarkers, Cancer genetics, Ovarian cancer

## Abstract

Somatic mutation of the tumor suppressor gene *TP53* is reported in at least 50% of human malignancies. Most high-grade serous ovarian cancers (HGSC) have a mutant *TP53* allele. Accurate detection of these mutants in heterogeneous tumor tissue is paramount as therapies emerge to target mutant p53. We used a Fluidigm Access Array™ System with Massively Parallel Sequencing (MPS) to analyze DNA extracted from 76 serous ovarian tumors. This dataset has been made available to researchers through the European Genome-phenome Archive (EGA; EGAS00001002200). Herein, we present analyses of this dataset using HaplotypeCaller and MuTect2 through the Broad Institute’s Genome Analysis Toolkit (GATK). We anticipate that this *TP53* mutation dataset will be useful to researchers developing and testing new software to accurately determine high and low frequency variant alleles in heterogeneous aneuploid tumor tissue. Furthermore, the analysis pipeline we present provides a valuable framework for determining somatic variants more broadly in tumor tissue.

## Background & Summary

The tumor suppressor gene *TP53* is the most frequently mutated gene in somatic cells of human cancers, with mutant *TP53* identified in over 50% of tumors^1,2,3,4,5^. While wild-type p53 acts to suppress a tumorigenic phenotype, both loss-of-function and oncogenic gain-of-function (GOF) *TP53* mutations promote tumorigenesis. In some tumors, such as high-grade serous ovarian cancers (HGSCs) *TP53* mutation is an early event, likely occurring in precursor lesions^6,7,8,9^. In colorectal cancer, mutation of *TP53* can occur as a relatively late event in a multistep tumorigenic pathway that progresses from hyperproliferative cells in colonic epithelium, through colorectal adenomas and finally metastatic colorectal cancer^10,11^. Germline mutation of *TP53* is associated with Li-Fraumeni syndrome where carriers are predisposed to develop malignancies including early onset breast cancer, brain and adrenocortical tumours, leukemia and soft tissue sarcoma^12^. Whether a mutation occurs in a single allele in the germline associated with increased risk of familial syndromes, or in sporadic cancers in somatic tissue where timing of its emergence may be different along the tumor progression pathway, has the potential to influence its frequency in tumor tissue.

There is a large and growing interest in targeting mutant p53 for cancer therapy^13,14,15^, resulting in a concomitant need to accurately detect the presence of a *TP53* mutation. This was the driving motivation for the original manuscript, i.e., to develop methodologies to accurately identify somatic *TP53* mutation in HGSC that could be used to triage women with this malignancy into appropriate trials targeting specific forms of mutant p53^16^. While the detection of a germline mutation in DNA extracted from a blood sample is relatively straightforward using the established method of Sanger sequencing, the detection of somatic DNA variants in tumor tissue, especially those occurring at low frequency, can pose challenges. Reasons for this include the heterogeneous nature of tumor tissue as the result of expansion of clonal populations and factors such as the presence of non-neoplastic cells, as well as aneuploidy, originating from tumor-associated phenomenon such as chromosomal instability^17^.

Massively parallel sequencing (MPS) of tumor tissue for variant detection in single genes of interest to the exclusion of either a cohort of other genes or the entire genome, is not broadly supported by current technologies in a cost effective manner. The Fluidigm Access Array System, specifically the Access Array BRCA1/BRCA2/TP53 Target-Specific Panel (Fluidigm, South San Francisco, CA, USA) coupled with MPS, was a cost effective way to achieve our goal of generating comprehensive MPS data for *TP53* in DNA extracted from a moderately sized cohort of primary ovarian tumors.

**Figure 1:**
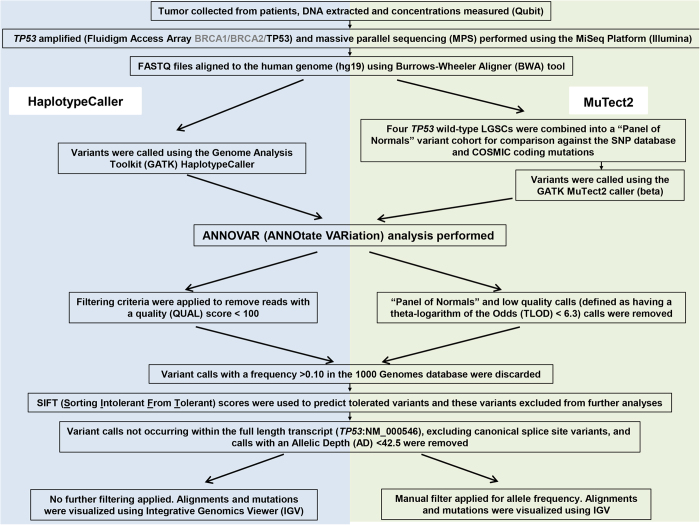
Overview of workflow and bioinformatic pipelines employed in this study.

Here, we present a detailed analysis of *TP53* MPS data using two software programs embedded within the Broad Institute’s Genome Analysis Toolkit (GATK), specifically HaplotypeCaller and MuTect2. HaplotypeCaller was specifically designed for the detection of germline mutations, although numerous papers have used this software for somatic variant calling^16,18,19,20,21^. MuTect2 has been designed to detect a range of variant allele frequencies, as described below. *TP53* variants identified by HaplotypeCaller were also screened for using Sanger sequencing and this data is presented. A schematic overview of this study, including bioinformatic pipelines, is presented (Fig. 1).

The dataset described herein offers a cohort on which to further develop robust methodologies and pipelines for determining a range of frequencies of somatic variants in tumor tissue that, by its very nature, is often heterogeneous and driven by genomic events resulting in aneuploidy. Data has been generated using DNA extracted from a cohort of HGSC (*N*=72) that is recognized as a genomically complex malignancy with extensive chromosomal abnormalities^22^. Given that a large study from The Cancer Genome Atlas (TCGA) reported over 96% of HGSC with a somatic *TP53* mutation^22^, there was an expectation that *TP53* mutation should be identified in the vast majority of HGSCs in this cohort. Somatic *TP53* mutation is not a feature of low-grade serous ovarian cancers (LGSC)^23^, of which four are included here and in the original study^16^.

## Methods

This section includes, and expands upon, the Methods outlined in our earlier manuscript^16^. When reference is made to previously published figures or tables (including Supplementary Data), the identifier is preceded by ‘OM’ denoting from the ‘Original Manuscript’. Methods, samples and datasets are outlined in the Experimental Study Table.

### Study cohort

Seventy-two HGSCs and four LGSCs collected from between 2004–2014 at three hospitals (Royal North Shore Hospital, North Shore Private and The Mater Hospital—North Sydney, Sydney, Australia) were analyzed for this study (Supplementary Table OM-S3). Advanced stage HGSCs (Stage III or IV) made up the majority of this cohort (82%; 59/72). Written informed patient consent was obtained as per our ethics protocol (Protocol: 108–243 M, approved by the Northern Sydney Local Health District Human Research Ethics Committee). All tumors were snap frozen in liquid nitrogen and stored in the Kolling Institute of Medical Research (KIMR) Gynecological Tumor Bank until required.

### Tumor DNA preparation

DNA was extracted from approximately 30 mg of fresh frozen tumor tissue. Tissue was homogenised in 50 μl phosphate buffered saline (PBS) until liquefied using two glass beads with shaking three times for 90 s each time at the highest frequency in a Retsch MM 301 Mixer Mill (MEP Instruments Pty. Ltd., NSW, Australia). Protein was digested at 56 °C overnight with 20 μl of proteinase K (20 mgml^−1^) (Qiagen Pty Ltd, Chadstone, VIC, Australia). DNA was extracted using the DNeasy Blood and Tissue Kit in an automated system (QIAcube; Qiagen Pty Ltd, Chadstone, VIC, Australia). DNA concentration was determined using Qubit Fluorometric Quantitation, specifically using the Qubit dsDNA BR Assay Kit (Life Technologies Australia Pty. Ltd., Mulgrave, VIC, Australia). A NanoDrop ND-1000 spectrophotometer (Thermo Fisher Scientific Australia, Scoresby, VIC, Australia) was used to determine 260:280 and 260:230 ratios.

### Fluidigm access array and massively parallel sequencing (MPS) of tumor DNA to identify somatic *TP53* mutations

As described in the original manuscript^16^, DNA extracted from tumors was processed for MPS using the Access Array BRCA1/BRCA2/TP53 Target-Specific Panel (Fluidigm, South San Francisco, CA, USA). The 48.48 Access Array integrated fluidic circuits (IFC) was used, including target specific primers containing a common sequence tag (CS1 or CS2) and Illumina adaptors PE1 and PE2. Samples were identified by a sample specific barcode located on the reverse sequence (PE1_CS1 Forward Primer, 5′- AATGATACGGCGACCACCGAGATCTACACTGACGACATGGTTCTACA-3′, 47 bp; PE2_BC_CS2 Reverse primer, 5′- CAAGCAGAAGACGGCATACGAGAT [sample specific barcode] TACGGTAGCAGAGACTTGGTCT-3′, 56 bp). This system uses 16 primer pairs generating amplicons of between 191–209 base pairs to enable 92% coverage of *TP53* exons.

Five μl of DNA (50 ngμl^−1^) was added to the array and processed on the Fluidigm Biomark HD Real-Time PCR fluidics system according to the manufacturer’s guidelines by the Ramaciotti Centre for Genomics (University of New South Wales, Randwick, Australia). Amplicon libraries were pooled and a single MPS run was performed on a MiSeq platform using Miseq Control Software (MCS) version 2.4.1 (Illumina Inc., San Diego, CA, USA).

### MPS data analysis and processing with HaplotypeCaller software

Sequencing data was received in FASTQ file format and adaptors trimmed using cutadapt (http://cutadapt.readthedocs.io/en/stable/guide.html). Trimmed FASTQ files were then aligned to the human genome (hg19) using Burrows-Wheeler Aligner (BWA) 0.7.10 and ‘known-indel’ realignment and recalibration which is embedded in the Broad Institute’s Genome Analysis Toolkit (GATK) Queue 3.2–2 data processing pipeline. The *TP53* gene region (chr17:7,569,720–7,592,868) was extracted from BAM files using samtools (http://samtools.sourceforge.net). At the time of publication of the original manuscript^16^, HaplotypeCaller was the variant analysis software embedded into the GATK best practice pipeline (GATK 3.2–2; https://www.broadinstitute.org/gatk/guide/best-practices). HaplotypeCaller assumes that DNA is from a diploid organism. It is best suited to germline variant calling; however, is able to detect allele frequencies outside of an expected 50:50 ratio. Annotation of variant calls was performed using ANNOVAR, version 2013J^24^.

Each sample summary was imported into Excel and filtered to display *TP53* variants, excluding intronic variants other than the canonical splice sites. Filtering criteria were applied to remove reads with a quality (QUAL) score less than 100. *TP53* variants were further filtered based on their frequency in the 1,000 Genome Database (Phase 3 integrated, all population, updated August2015)^25^. If a particular variant occurred at a frequency greater than 10% in this database, the variant was deemed to be non-deleterious and excluded from the analysis. Lastly, variants were filtered based on SIFT scores (Sorting Intolerant From Tolerant; from dbNSFP v3.0 that amalgamates SIFT to the version based on Ensembl 66. For release 66, Ensembl ran SIFT version 4.0.5 using UniProtKB [release 2012_01, both the SwissProt and TrEMBL sets]). SIFT is an *in silico* tool for predicting the functional effects of a variant on the associated protein^26^. Variants predicted to be tolerated were excluded. All remaining variants were considered deleterious or did not have a SIFT score and were visualized using the Integrative Genomics Viewer (IGV, v2.3, www.broadinstitute.org)^27,28^. The allele frequency of each mutation was recorded upon visualization of the mutation *via* IGV. This analysis pipeline was previously summarized (Supplementary Fig. OM-S5).

### MPS data processing and analysis with MuTect2 software

Since publication of the original manuscript^16^, MuTect2 has become available through GATK that combines aspects of the original MuTect^29^ and HaplotypeCaller for somatic genotyping. MuTect2 detects a range of allele frequencies, making it eminently more suitable for somatic genotyping in heterogeneous, often aneuploid, tumor tissue compared to HaplotypeCaller that was designed for germline variant calling where alleles are present in equal ratios. FASTQ files were trimmed and aligned as described for HaplotypeCaller. Somatic variant calling was performed using MuTect2 beta in GATK version 3.6. The four LGSCs (previously shown to be wild-type for *TP53* using identical MPS and analysis pipelines to the HGSCs studied; Supplementary Table OM-S2) were combined into a Panel of Normals (PoN) variant cohort against the Single Nucelotide Polymorphism database current build 138 (dbsnp138) and COSMIC coding mutations. Tumor only variant-calling was then performed using the pre-generated PoN for each tumor sample. MuTect2 software requires a minimum of two samples to create a PoN variant call format (VCF) file. Each tumor VCF was annotated using ANNOVAR (2016Feb01; http://annovar.openbioinformatics.org/en/latest/) and merged into an Excel spreadsheet for downstream analyses.

The PoN calls were removed, as were low quality calls (defined as having a theta-logarithm of the Odds (TLOD)<6.3). Synonymous variant calls were filtered out along with variants in intronic and untranslated regions. Non-deleterious calls were filtered out based on SIFT scores as above. Variant calls not occurring within the full length transcript (TP53:NM_000546) or canonical splice sites were also removed. Lastly, a manual filter was applied to remove variant calls occurring at a frequency of less than 5%.

### Code availability

All tools required for the analysis of this data are freely available. Instructions for downloading and installation are in scripts.sh (https://figshare.com/articles/scripts_sh/4542397).

1. wget to retrieve BAM files (binary version of tab-delimited text files containing sequence alignment data and the recommended format for IGV) from the online EGA web server that has archived this data.
2. GATK for somatic variant calling in tumor samples can be performed using Mutect2 as part of the GATK pipeline.
3. ANNOVAR to annotate variant information to prioritize somatic variant calling.

The requirements for running GATK and ANNOVAR can be referenced from each website respectively. Analysis scripts (bash shell code) should be run in the MacOS/Unix system by opening ~/Applications/Utilities/Terminal.app.

For re-analysis of data, registration will be required for GATK version 3.6 (https://figshare.com/articles/GenomeAnalysisTK_jar/4541719) and ANNOVAR (2016Feb01). The file named ‘script.sh’ (https://figshare.com/articles/scripts_sh/4542397) will need to be downloaded in which the section uses ‘/path/to/’ in order to indicate paths that should be modified by the user depending on the location the data files are to be downloaded to. Certain files will require download as compressed files that will need decompression and setting of a path to the executable file. Script pipelines may take 22 h to run on a 4 cores, 16 GB personal computer. All file downloads will require 34 GB of storage space.

## Data Record

*TP53* MPS data (Data Citation 1) is available in the European Genome-phenome Archive (EGA) with the study accession number EGAS00001002200 and dataset accession number EGAD00001003119 (Table 1 (available online only)). This dataset contains MPS information on 76 unique tumor samples from individual patients, of which four are LGSCs and 72 are HGSCs. All sample files are in the BAM format and have been extracted to have the p53 gene (*TP53*) region reads along with the unmapped reads.

## Technical Validation

### Quality control—assessment of percentage tumor cells in each sample

A pathologist [AJG] reviewed all tumor tissue in order to confirm diagnosis, histological grade and pathological stage. Sequential sections from frozen tumors were analyzed to determine percent tumour cells after staining with hematoxylin and eosin. For inclusion in this study, tumors were required to contain a minimum of 5% tumor cells. The percent tumor cell composition in samples used in this study ranged from 5–90% (Supplementary Table OM-S1).

### Quality control—DNA integrity

Prior to analysis on the Fluidigm Access Array, DNA integrity was assessed using the Qubit dsDNA BR Assay Kit for fluorimetric quantitation. This assay is selective for double-stranded DNA (dsDNA) over RNA and is designed for optimal performance within a concentration range of 100 pg–1,000 ngμl^−1^. Based on this quantitation, DNA was diluted to 50 ngμl^−1^ using nuclease and PCR inhibitor free elution buffer EA from the QIAamp DNA Mini Kit (Qiagen Pty Ltd). DNA was confirmed to be clean by assessment of 260:280 and 260:230 ratios >1.8 using the NanoDrop.

### Quality control -massively parallel sequencing (MPS) data and analysis

As described in the original manuscript, amplicon libraries for 72 samples were generated using the Access Array BRCA1/BRCA2/TP53 Target-Specific Panel (Fluidigm, South San Francisco, CA, USA), pooled and sequenced in a single run on a MiSeq platform using Miseq Control Software (MCS) version 2.4.1 (Illumina Inc., San Siego, CA, USA)^16^. This single sequencing run produced a cluster density of 1,133±31 K/MM^2^ (84.57%±1.89 passing filter) and 20,626,284 sequence reads (17,452,900 passing filter) with 95.42% ≥Q30 (Read 1) and 92.61% ≥Q30 (Read 2).

**Figure 2:**
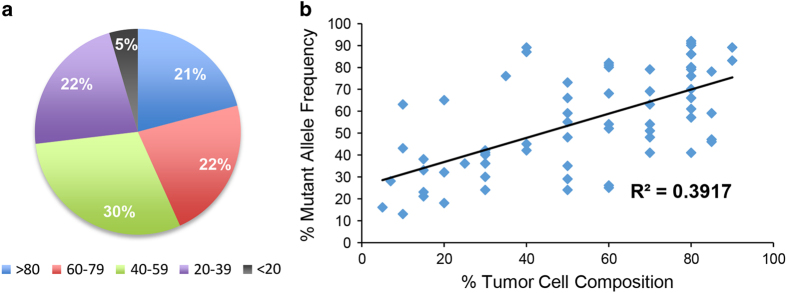
Mutant alleles identified by HaplotypeCaller. (**a**) Schematic representation of the proportion of tumor samples in our cohort in which different frequencies of variant alleles were detected. Color coding represents the frequency of variant alleles within individual samples. (**b**) Correlation between mutant allele frequency in tumor tissue and percent tumor cell composition. Tumor percentage was plotted against *TP53* mutant allele frequency for 67 HGSC samples and the R^2^ value determined (R^2^=0.3917).

Described in methods, analysis of MPS data, using both HaplotypeCaller and MuTect2, required extensive filtering to remove reads of poor quality, variants that appeared in datasets of normal genomes and variants predicted to be non-pathogenic. Filtering protocols are summarized in Fig. 1 as part of our bioinformatics analysis pipeline. The allele frequency for *TP53* mutations called by MPS using HaplotypeCaller ranged from 13–92% in the HGSC cohort (mean and median values 55 and 54% respectively; Supplementary Table OM-S1 and Fig. 2a). The *TP53* mutant allele frequency for a single sample with a large in-frame insertion (#880–13 [c.723_724dupACCATCCACTACAACTACATGTGTAACAGTTCC]; Supplementary Table OM-S1) was unable to be determined with our analysis pipeline, although was detectable by Sanger sequencing.

We assessed whether the percent tumor cell composition was likely to influence the frequency of the mutant alleles that were detected. These two variables were graphed, a line of best fit plotted and the R^2^ value calculated (Fig. 2b). This analysis demonstrated a small correlation (R^2^=0.3917) between these two variables, suggesting that our minimum criteria of 5% tumor cell composition was adequate for detecting *TP53* variants using our pipeline. Any concerns regarding potential influence of a low percentage of tumor cells could be circumvented by the use of tumor macro- or micro-dissection to ensure a more pure cancer cell population for analysis^30^.

Re-analysis of our data with MuTect2 (beta) resulted in identification of all of the variants detected by HaplotypeCaller, with one exception discussed below, and an additional five *TP53* variants with allele frequencies ranging from approximately 2–3% (Table 2). We excluded these variants by setting a manual filter for all frequencies below 5%. It is unclear whether these low frequency variants are artefacts introduced by MuTect2 software, or indeed represent very low frequency somatic *TP53* mutations in sporadic tumors. We do not have further access to these specimens to investigate them with alternative methodology such as digital PCR that may detect very low frequency variant alleles. If these low frequency variants are not artefacts, their biological significance in the tumor milieu is unclear. The possibility that MuTect2 software can detect very low frequency alleles in heterogeneous cell populations may be of relevance in some malignancies where active screening for early relapse and/or response to therapy is undertaken. Furthermore, analyses with MuTect2 showed the allele frequency of the large in-frame insertion (#880-13; Supplementary Table OM-S1) as 0.5%. Given that we could easily visualize this mutation using Sanger sequencing that we showed in the original manuscript^16^ could not reliably detect variants at allele frequencies less than 25%, it is not possible that this insertion is present at such a low frequency in this tumor. This data suggests that care should be taken when using MuTect2 to identify variants involving larger alterations.

## Usage Notes

The following gives clear instructions as to how to apply to access the dataset described in this manuscript.

Use the search bar on the front page of the European Genome-Phenome Archive website (https://ega-archive.org/) to search for this study with a keyword such as ‘TP53’ or the study ID number that is EGAS00001002200. This will bring you to a screen where you can view information on datasets, data providers, data access committees (DACs) and any other documentation associated with this study. A description of this study is located under the heading ‘Study Description’. There is a single dataset associated with this study (Study ‘Datasets 1 dataset’ and its data ID number is EGAD0001003119. Click on this dataset ID to take you to information about who to contact regarding access to this data.

Each dataset in EGA is affiliated to a Data Access Committee (DAC), which is the group responsible for data access decisions following a formal application process. Access to actual data files is not managed by the EGA. You must apply to this DAC to gain access to this controlled dataset using your EGA account. Upon clicking on the dataset ID, you will come to the heading ‘Who controls access to this dataset’. For requests to access this dataset, please contact:

**DAC:** Functional Genomics Laboratory, Kolling Institute of Medical Research DAC—TP53 mutation data in ovarian cancer.

**Contact Person**: Deborah Marsh

**Email:** deborah [dot] marsh [at] Sydney [dot] edu [dot] au

**More details:** EGAC00001000589

A Data Access Agreement (DAA) will be required. The DAA is a contract between the proposed user of the data and the DAC. This will contain information such as details of data use, publication embargoes and storage of data. Completion of a DAA by the applicant(s) should be considered as part of the application process to the DAC. A template DAA can be found on the EGA website under ‘Policy documentation—Data Access Agreement (DAA)’. A modified template specific for this dataset is provided as Supplementary Data. The completed EGA DAA signed by both parties (the data provider and those wishing to access the data) should be emailed to ega-helpdesk@ebi.ac.uk.

Upon receiving the completed DAA approved by the DAC, EGA will arrange a one-time login to set a password for your EGA account that will be sent to your email address. Following authorisation of your password, you will receive email notification that your EGA account is ready for your use. A list of the datasets you have been granted access to will appear on your ‘My Datasets’ page in EGA. From here, you will be able to download the data.

## Additional Information

**How to cite this article:** Cole, A. J. *et al.* Comprehensive analyses of somatic *TP53* mutation in tumors with variable mutant allele frequency. *Sci. Data* 4:170120 doi: 10.1038/sdata.2017.120 (2017).

**Publisher’s note:** Springer Nature remains neutral with regard to jurisdictional claims in published maps and institutional affiliations.

## Supplementary Material



## Figures and Tables

**Table 1:** Experimental Study Table

**Sample ID**	**Tumor Type**	**Protocol 1**	**Protocol 2**	**Protocol 3**	**Study Accession Number** *	**Dataset Accession Number** *
42470	HGSC†	Tumor DNA extraction	Fluidigm Access Array™ BRCA1/BRCA2/TP53	Massively Parallel Sequencing (MiSeq)	EGAS00001002200	EGAD00001003119
42474	HGSC	Tumor DNA extraction	Fluidigm Access Array™ BRCA1/BRCA2/TP53	Massively Parallel Sequencing (MiSeq)	EGAS00001002200	EGAD00001003119
42478	HGSC	Tumor DNA extraction	Fluidigm Access Array™ BRCA1/BRCA2/TP53	Massively Parallel Sequencing (MiSeq)	EGAS00001002200	EGAD00001003119
40-05	HGSC	Tumor DNA extraction	Fluidigm Access Array™ BRCA1/BRCA2/TP53	Massively Parallel Sequencing (MiSeq)	EGAS00001002200	EGAD00001003119
47-05	HGSC	Tumor DNA extraction	Fluidigm Access Array™ BRCA1/BRCA2/TP53	Massively Parallel Sequencing (MiSeq)	EGAS00001002200	EGAD00001003119
64-05	HGSC	Tumor DNA extraction	Fluidigm Access Array™ BRCA1/BRCA2/TP53	Massively Parallel Sequencing (MiSeq)	EGAS00001002200	EGAD00001003119
74-05	HGSC	Tumor DNA extraction	Fluidigm Access Array™ BRCA1/BRCA2/TP53	Massively Parallel Sequencing (MiSeq)	EGAS00001002200	EGAD00001003119
76-05	HGSC	Tumor DNA extraction	Fluidigm Access Array™ BRCA1/BRCA2/TP53	Massively Parallel Sequencing (MiSeq)	EGAS00001002200	EGAD00001003119
105-06	HGSC	Tumor DNA extraction	Fluidigm Access Array™ BRCA1/BRCA2/TP53	Massively Parallel Sequencing (MiSeq)	EGAS00001002200	EGAD00001003119
106-06	HGSC	Tumor DNA extraction	Fluidigm Access Array™ BRCA1/BRCA2/TP53	Massively Parallel Sequencing (MiSeq)	EGAS00001002200	EGAD00001003119
109-06	HGSC	Tumor DNA extraction	Fluidigm Access Array™ BRCA1/BRCA2/TP53	Massively Parallel Sequencing (MiSeq)	EGAS00001002200	EGAD00001003119
94-06	HGSC	Tumor DNA extraction	Fluidigm Access Array™ BRCA1/BRCA2/TP53	Massively Parallel Sequencing (MiSeq)	EGAS00001002200	EGAD00001003119
99-06	HGSC	Tumor DNA extraction	Fluidigm Access Array™ BRCA1/BRCA2/TP53	Massively Parallel Sequencing (MiSeq)	EGAS00001002200	EGAD00001003119
123-07	HGSC	Tumor DNA extraction	Fluidigm Access Array™ BRCA1/BRCA2/TP53	Massively Parallel Sequencing (MiSeq)	EGAS00001002200	EGAD00001003119
133-07	HGSC	Tumor DNA extraction	Fluidigm Access Array™ BRCA1/BRCA2/TP53	Massively Parallel Sequencing (MiSeq)	EGAS00001002200	EGAD00001003119
156-07	HGSC	Tumor DNA extraction	Fluidigm Access Array™ BRCA1/BRCA2/TP53	Massively Parallel Sequencing (MiSeq)	EGAS00001002200	EGAD00001003119
157-07	HGSC	Tumor DNA extraction	Fluidigm Access Array™ BRCA1/BRCA2/TP53	Massively Parallel Sequencing (MiSeq)	EGAS00001002200	EGAD00001003119
198-08	HGSC	Tumor DNA extraction	Fluidigm Access Array™ BRCA1/BRCA2/TP53	Massively Parallel Sequencing (MiSeq)	EGAS00001002200	EGAD00001003119
206-08	HGSC	Tumor DNA extraction	Fluidigm Access Array™ BRCA1/BRCA2/TP53	Massively Parallel Sequencing (MiSeq)	EGAS00001002200	EGAD00001003119
230-08	HGSC	Tumor DNA extraction	Fluidigm Access Array™ BRCA1/BRCA2/TP53	Massively Parallel Sequencing (MiSeq)	EGAS00001002200	EGAD00001003119
237-08	HGSC	Tumor DNA extraction	Fluidigm Access Array™ BRCA1/BRCA2/TP53	Massively Parallel Sequencing (MiSeq)	EGAS00001002200	EGAD00001003119
309-09	HGSC	Tumor DNA extraction	Fluidigm Access Array™ BRCA1/BRCA2/TP53	Massively Parallel Sequencing (MiSeq)	EGAS00001002200	EGAD00001003119
337-09	HGSC	Tumor DNA extraction	Fluidigm Access Array™ BRCA1/BRCA2/TP53	Massively Parallel Sequencing (MiSeq)	EGAS00001002200	EGAD00001003119
353-09	HGSC	Tumor DNA extraction	Fluidigm Access Array™ BRCA1/BRCA2/TP53	Massively Parallel Sequencing (MiSeq)	EGAS00001002200	EGAD00001003119
381-09	HGSC	Tumor DNA extraction	Fluidigm Access Array™ BRCA1/BRCA2/TP53	Massively Parallel Sequencing (MiSeq)	EGAS00001002200	EGAD00001003119
416-09	HGSC	Tumor DNA extraction	Fluidigm Access Array™ BRCA1/BRCA2/TP53	Massively Parallel Sequencing (MiSeq)	EGAS00001002200	EGAD00001003119
466-10	HGSC	Tumor DNA extraction	Fluidigm Access Array™ BRCA1/BRCA2/TP53	Massively Parallel Sequencing (MiSeq)	EGAS00001002200	EGAD00001003119
471-10	HGSC	Tumor DNA extraction	Fluidigm Access Array™ BRCA1/BRCA2/TP53	Massively Parallel Sequencing (MiSeq)	EGAS00001002200	EGAD00001003119
472-10	HGSC	Tumor DNA extraction	Fluidigm Access Array™ BRCA1/BRCA2/TP53	Massively Parallel Sequencing (MiSeq)	EGAS00001002200	EGAD00001003119
490-10	HGSC	Tumor DNA extraction	Fluidigm Access Array™ BRCA1/BRCA2/TP53	Massively Parallel Sequencing (MiSeq)	EGAS00001002200	EGAD00001003119
497-10	HGSC	Tumor DNA extraction	Fluidigm Access Array™ BRCA1/BRCA2/TP53	Massively Parallel Sequencing (MiSeq)	EGAS00001002200	EGAD00001003119
521-10	HGSC	Tumor DNA extraction	Fluidigm Access Array™ BRCA1/BRCA2/TP53	Massively Parallel Sequencing (MiSeq)	EGAS00001002200	EGAD00001003119
531-10	HGSC	Tumor DNA extraction	Fluidigm Access Array™ BRCA1/BRCA2/TP53	Massively Parallel Sequencing (MiSeq)	EGAS00001002200	EGAD00001003119
537-10	HGSC	Tumor DNA extraction	Fluidigm Access Array™ BRCA1/BRCA2/TP53	Massively Parallel Sequencing (MiSeq)	EGAS00001002200	EGAD00001003119
543-10	HGSC	Tumor DNA extraction	Fluidigm Access Array™ BRCA1/BRCA2/TP53	Massively Parallel Sequencing (MiSeq)	EGAS00001002200	EGAD00001003119
565-10	HGSC	Tumor DNA extraction	Fluidigm Access Array™ BRCA1/BRCA2/TP53	Massively Parallel Sequencing (MiSeq)	EGAS00001002200	EGAD00001003119
568-10	HGSC	Tumor DNA extraction	Fluidigm Access Array™ BRCA1/BRCA2/TP53	Massively Parallel Sequencing (MiSeq)	EGAS00001002200	EGAD00001003119
586-11	HGSC	Tumor DNA extraction	Fluidigm Access Array™ BRCA1/BRCA2/TP53	Massively Parallel Sequencing (MiSeq)	EGAS00001002200	EGAD00001003119
612-11	HGSC	Tumor DNA extraction	Fluidigm Access Array™ BRCA1/BRCA2/TP53	Massively Parallel Sequencing (MiSeq)	EGAS00001002200	EGAD00001003119
614-11	HGSC	Tumor DNA extraction	Fluidigm Access Array™ BRCA1/BRCA2/TP53	Massively Parallel Sequencing (MiSeq)	EGAS00001002200	EGAD00001003119
630-11	HGSC	Tumor DNA extraction	Fluidigm Access Array™ BRCA1/BRCA2/TP53	Massively Parallel Sequencing (MiSeq)	EGAS00001002200	EGAD00001003119
631-11	HGSC	Tumor DNA extraction	Fluidigm Access Array™ BRCA1/BRCA2/TP53	Massively Parallel Sequencing (MiSeq)	EGAS00001002200	EGAD00001003119
634-11	HGSC	Tumor DNA extraction	Fluidigm Access Array™ BRCA1/BRCA2/TP53	Massively Parallel Sequencing (MiSeq)	EGAS00001002200	EGAD00001003119
638-11	HGSC	Tumor DNA extraction	Fluidigm Access Array™ BRCA1/BRCA2/TP53	Massively Parallel Sequencing (MiSeq)	EGAS00001002200	EGAD00001003119
651-11	HGSC	Tumor DNA extraction	Fluidigm Access Array™ BRCA1/BRCA2/TP53	Massively Parallel Sequencing (MiSeq)	EGAS00001002200	EGAD00001003119
666-11	HGSC	Tumor DNA extraction	Fluidigm Access Array™ BRCA1/BRCA2/TP53	Massively Parallel Sequencing (MiSeq)	EGAS00001002200	EGAD00001003119
676-11	HGSC	Tumor DNA extraction	Fluidigm Access Array™ BRCA1/BRCA2/TP53	Massively Parallel Sequencing (MiSeq)	EGAS00001002200	EGAD00001003119
679-11	HGSC	Tumor DNA extraction	Fluidigm Access Array™ BRCA1/BRCA2/TP53	Massively Parallel Sequencing (MiSeq)	EGAS00001002200	EGAD00001003119
694-11	HGSC	Tumor DNA extraction	Fluidigm Access Array™ BRCA1/BRCA2/TP53	Massively Parallel Sequencing (MiSeq)	EGAS00001002200	EGAD00001003119
702-11	HGSC	Tumor DNA extraction	Fluidigm Access Array™ BRCA1/BRCA2/TP53	Massively Parallel Sequencing (MiSeq)	EGAS00001002200	EGAD00001003119
711-11	HGSC	Tumor DNA extraction	Fluidigm Access Array™ BRCA1/BRCA2/TP53	Massively Parallel Sequencing (MiSeq)	EGAS00001002200	EGAD00001003119
764-12	HGSC	Tumor DNA extraction	Fluidigm Access Array™ BRCA1/BRCA2/TP53	Massively Parallel Sequencing (MiSeq)	EGAS00001002200	EGAD00001003119
767-12	HGSC	Tumor DNA extraction	Fluidigm Access Array™ BRCA1/BRCA2/TP53	Massively Parallel Sequencing (MiSeq)	EGAS00001002200	EGAD00001003119
778-12	HGSC	Tumor DNA extraction	Fluidigm Access Array™ BRCA1/BRCA2/TP53	Massively Parallel Sequencing (MiSeq)	EGAS00001002200	EGAD00001003119
787-12	HGSC	Tumor DNA extraction	Fluidigm Access Array™ BRCA1/BRCA2/TP53	Massively Parallel Sequencing (MiSeq)	EGAS00001002200	EGAD00001003119
849-13	HGSC	Tumor DNA extraction	Fluidigm Access Array™ BRCA1/BRCA2/TP53	Massively Parallel Sequencing (MiSeq)	EGAS00001002200	EGAD00001003119
862-13	HGSC	Tumor DNA extraction	Fluidigm Access Array™ BRCA1/BRCA2/TP53	Massively Parallel Sequencing (MiSeq)	EGAS00001002200	EGAD00001003119
879-13	HGSC	Tumor DNA extraction	Fluidigm Access Array™ BRCA1/BRCA2/TP53	Massively Parallel Sequencing (MiSeq)	EGAS00001002200	EGAD00001003119
880-13	HGSC	Tumor DNA extraction	Fluidigm Access Array™ BRCA1/BRCA2/TP53	Massively Parallel Sequencing (MiSeq)	EGAS00001002200	EGAD00001003119
938-13	HGSC	Tumor DNA extraction	Fluidigm Access Array™ BRCA1/BRCA2/TP53	Massively Parallel Sequencing (MiSeq)	EGAS00001002200	EGAD00001003119
943-13	HGSC	Tumor DNA extraction	Fluidigm Access Array™ BRCA1/BRCA2/TP53	Massively Parallel Sequencing (MiSeq)	EGAS00001002200	EGAD00001003119
949-13	HGSC	Tumor DNA extraction	Fluidigm Access Array™ BRCA1/BRCA2/TP53	Massively Parallel Sequencing (MiSeq)	EGAS00001002200	EGAD00001003119
958-13	HGSC	Tumor DNA extraction	Fluidigm Access Array™ BRCA1/BRCA2/TP53	Massively Parallel Sequencing (MiSeq)	EGAS00001002200	EGAD00001003119
1001-14	HGSC	Tumor DNA extraction	Fluidigm Access Array™ BRCA1/BRCA2/TP53	Massively Parallel Sequencing (MiSeq)	EGAS00001002200	EGAD00001003119
1004-14	HGSC	Tumor DNA extraction	Fluidigm Access Array™ BRCA1/BRCA2/TP53	Massively Parallel Sequencing (MiSeq)	EGAS00001002200	EGAD00001003119
966-14	HGSC	Tumor DNA extraction	Fluidigm Access Array™ BRCA1/BRCA2/TP53	Massively Parallel Sequencing (MiSeq)	EGAS00001002200	EGAD00001003119
969-14	HGSC	Tumor DNA extraction	Fluidigm Access Array™ BRCA1/BRCA2/TP53	Massively Parallel Sequencing (MiSeq)	EGAS00001002200	EGAD00001003119
985-14	HGSC	Tumor DNA extraction	Fluidigm Access Array™ BRCA1/BRCA2/TP53	Massively Parallel Sequencing (MiSeq)	EGAS00001002200	EGAD00001003119
881-13	HGSC	Tumor DNA extraction	Fluidigm Access Array™ BRCA1/BRCA2/TP53	Massively Parallel Sequencing (MiSeq)	EGAS00001002200	EGAD00001003119
695-11	HGSC	Tumor DNA extraction	Fluidigm Access Array™ BRCA1/BRCA2/TP53	Massively Parallel Sequencing (MiSeq)	EGAS00001002200	EGAD00001003119
493-10	HGSC	Tumor DNA extraction	Fluidigm Access Array™ BRCA1/BRCA2/TP53	Massively Parallel Sequencing (MiSeq)	EGAS00001002200	EGAD00001003119
427-09	HGSC	Tumor DNA extraction	Fluidigm Access Array™ BRCA1/BRCA2/TP53	Massively Parallel Sequencing (MiSeq)	EGAS00001002200	EGAD00001003119
544-10	LGSC‡	Tumor DNA extraction	Fluidigm Access Array™ BRCA1/BRCA2/TP53	Massively Parallel Sequencing (MiSeq)	EGAS00001002200	EGAD00001003119
624-11	LGSC	Tumor DNA extraction	Fluidigm Access Array™ BRCA1/BRCA2/TP53	Massively Parallel Sequencing (MiSeq)	EGAS00001002200	EGAD00001003119
909-13	LGSC	Tumor DNA extraction	Fluidigm Access Array™ BRCA1/BRCA2/TP53	Massively Parallel Sequencing (MiSeq)	EGAS00001002200	EGAD00001003119
730-12	LGSC	Tumor DNA extraction	Fluidigm Access Array™ BRCA1/BRCA2/TP53	Massively Parallel Sequencing (MiSeq)	EGAS00001002200	EGAD00001003119

*Data can be accessed through the European Genome-phenome Archive (EPA).

^†^human high-grade serous ovarian cancer.

^‡^human low-grade serous ovarian cancer.

**Table 2:** Additional *TP53* variants identified by MuTect2 at low frequency.

**Sample ID**	**Genomic position (chr:start-end)**	**Reference: Variant allele (%)**	**Exon**	**cDNA change**	**Protein effect**	**Reference: Variant allele read count**	**Tumor variant allele ratio**	**SIFT call**	Database Presence (IARC*)	**% Tumor cell composition**
10-04	17:7578440–7578440	T(98%): C(2%)	5	c.490A>G	Lys164Glu	2929:58	0.02	D	Yes	70
198-08	17:7577541–7577541	T(97%): C(3%)	7	c.740A>G	Asn247Ser	2336:60	0.026	D	Yes	10
198-08	17:7579471–7579471	G(97%): −(3%)	4	c.216delC	Pro72Argfs*49	2116:56	0.026	N/A	No	10
206-08	17:7577556–7577556	C(98%): G(2%)	7	c.725G>C	Cys242Ser	2334:53	0.023	D	Yes	50
427-09	17:7577574–7577574	T(97%): C(3%)	7	c.707A>G	Try236Cys	2003:59	0.029	D	Yes	80
^D, Deleterious; N/A, no SIFT call;										

*IARC, International Agency for Research on Cancer.
